# Supplementary material for: Content-rich biological network constructed by mining PubMed abstracts
Source: BMC Bioinformatics. 2004 Oct 8;5:147. doi: 10.1186/1471-2105-5-147 (PMC528731; doi:10.1186/1471-2105-5-147)
Supplement: Additional File 2 — The original results of the above study (non-essential files are deleted to keep the file size under the limit set by BMC bioinformatics). [file 1471-2105-5-147-S2.bz2 › chilibotAdditionalFile2/dip10/430ID7622448E10360/html/CCR1_CCL5.html]

 


 **CCR1** and **CCL5** 
  
Found 208 abstracts in PubMed, retrieved 10.  
 

 What does Google say? 
 PDF only 
| .edu only 

---

**Interactive relationship** (e.g. stimulation, inhibition, etc)

**Neutral relationship**- Oligomerization of RANTES  [ **CCL5** ]  is required for  **CCR1**  mediated arrest but not CCR5 mediated transmigration of leukocytes on inflamed endothelium.  Ref: 12763925 Blood< MedlineTA>Blood,
- Efficient leukocyte arrest in flow but not transmigration may thus require the presentation of RANTES  [ **CCL5** ]  oligomers to bridge surface bound RANTES  [ **CCL5** ]  and  **CCR1** .  Ref: 12763925 Blood< MedlineTA>Blood,
- Notably, the formation of higher order RANTES  [ **CCL5** ]  oligomers was crucial for  **CCR1**  mediated arrest but not for CCR5 mediated spreading transmigration in flow or transendothelial chemotaxis of leukocytes.  Ref: 12763925 Blood< MedlineTA>Blood,
- Expression of L CCR  [ **CCR1** ]  in HEK 293 cells reveals functional responses to CCL2,  **CCL5** , CCL7, and CCL8.  Ref: 12885941 J Leukoc Biol, 2003
- One such receptor is  **CCR1**  which is a receptor for CC chemokines, such as  **CCL5**  RANTES  [ **CCL5** ]  and CCL3 MIP 1alpha.  Ref: 12769747 Curr Pharm Des, 2003
- However, the remaining expression of  **CCR1**  and CCR5 correlated reciprocally with an ongoing unresponsiveness to the  **CCR1**  and CCR5 agonists CCL3 macrophage inflammatory protein 1alpha MIP 1alpha, CCL4 MIP 1beta, and  **CCL5**  regulated on activation, normal T expressed and secreted RANTES  [ **CCL5** ] , all chemokines binding to these two receptors.  Ref: 12885942 J Leukoc Biol, 2003
- However, the specific functional relevance of RANTES  [ **CCL5** ]  oligomerization for distinct steps of leukocyte recruitment on inflamed endothelium mediated by the RANTES  [ **CCL5** ]  receptors  **CCR1**  and CCR5 remains undefined.  Ref: 12763925 Blood< MedlineTA>Blood, 2003
- The present findings suggest that MCP 1, RANTES  [ **CCL5** ] , and IP 10 may participate in the pathogenesis of mercuric chloride induced tubulointerstitial fibrosis with mononuclear cell infiltration, via CCR2,  **CCR1**  or CCR5, and CXCR3, respectively.  Ref: 12834626 Exp Mol Pathol, 2003

**Non-interactive relationship** (e.g. studied together, co-existance, homology, etc.)

- To test the in vivo relevance of this, we used a murine melanoma system and knockout mice to investigate the function of the chemokine receptor CCR5 and its ligands, CCR  [ **CCR1** ]  ligand CCL 3 and  **CCL5** .  Ref: 12682253 J Immunol, 2003
- After 2 to 4 weeks, L. amazonensis infected mice had significantly delayed and depressed expression of inflammatory cytokines interleukin 12 IL 12, gamma interferon, IL 1alpha, IL 1beta, CC chemokines CC chemokine ligand 3 CCL3 macrophage inflammatory protein 1alpha MIP 1alpha, CCL4 MIP 1beta,  **CCL5**  RANTES  [ **CCL5** ] , MIP 2, and chemokine receptors  **CCR1** , CCR2, CCR5 in foot tissues and draining lymph nodes compared to the expression in L. major infected controls.  Ref: 12874303 Infect Immun, 2003
